# Rhizomicrobiomics

**DOI:** 10.1093/sumbio/qvae016

**Published:** 2024-07-13

**Authors:** Jim Lynch

**Affiliations:** Centre for Environment and Sustainability, University of Surrey, Guildford, Surrey GU2 7XH, United Kingdom

**Keywords:** rhizosphere, microbiome, biological control, food, bioremediation, climate change, rhizomicrobiomics

## Abstract

Rhizomicrobiomics is the study of plant-associated microbes as a strategy for achieving sustainable development goals. With the development of the concept of microbiomes of soil/plant systems, the history leading to this concept over more than a century is reviewed. Microbial growth and community dynamics are discussed from both laboratory and field perspectives. The first use of the term microbiome applied to biological control of plant diseases, but it now seems appropriate to use the specific term rhizomicrobiome to describe the myriads of microbial functions that influence soil health, food production, bioremediation, and climate change. The advance and implications of molecular biology and modern imaging, along with functional analysis of ecosystems from space, coupled with artificial intelligence and machine learning, are indicated as ways to investigate the application of rhizomicrobiomics in achieving the UN Sustainable Development Goals to generate a cleaner planet and secure the future supply of food.

**Sustainability Statement**

Much of the article is associated with UN Sustainable Development Goal SDG 15 (Life on Land) but is relevant to others: SDG 1 (No Poverty), SDG 2 (Zero Hunger), SDG 3 (Good Health and Well Being), and SDG 13 (Climate Action). In the UK, there is enthusiasm at many levels in the public and private sectors to improve soil health and use soil sustainably. Rhizomicrobiomics is an important component of the analysis of land use and land use change, which is a major sustainability issue internationally.

## Introduction

Some of the earliest critical studies of soil microorganisms were at the beginning of the 20th century. For example, Hiltner studied bacterial types in soils based on general groups rather than definite species and first termed their interactions with plants as the rhizosphere (Hiltner 1904). Mid-century studies in the Department of Agriculture at Ottawa, Canada, developed the quantitative analysis of such organisms as they related to plants in the rhizosphere (e.g. Lochhead and Taylor 1938, Lochhead and Thexton 1947). In the 1960s and beyond, such research activity was promoted at key research institutes such as the CSIRO Division of Soils in Adelaide, Australia, and the Agricultural and Food Research Council Letcombe Laboratory in Oxfordshire, England in considering the root-soil interface as critical for crop production. This research was summarized in books by Russell (1977) and Lynch (1990).

As soil microbiology developed from the 1960s, the study of microbial population and community dynamics developed in the laboratory in places such as the Institut Pasteur in Paris, led by Jacques Monod (1942), and Queen Elizabeth (now King’s) College in London, led by John Pirt (1975). These studies showed primarily how single species grew but also how stable mixed cultures could develop in a vessel known as a chemostat with a continuous substrate feed and outflow. Pirt’s book considered two species growing alongside each other in pure culture to reach stability, but of course, in nature, multiple species grow together to make a community to carry out function. Using traditional methods of isolation, a wide range of organisms can be identified, but many species, perhaps as many as 90%, are not culturable by these methods. With the rapid advancement of molecular techniques this century, most organisms can now be identified at both the cellular and functional levels. It is also important to recognize that microbial communities in the rhizosphere are composed of bacteria, archaea, fungi, and protozoa, all of which can interact to contribute to function. In addition, some of them will be highly dependent on the plant as symbionts or pathogens.

The behaviour of microorganisms in nature was facilitated with the rapid advance of the sub-discipline of microbial ecology in the 1970s and 1980s (Lynch and Poole 1979, Lynch and Hobbie 1988). During this time, *soil biotechnology* was also classified as the study of soil microorganisms and their metabolic processes to optimize crop productivity (Lynch 1983). Biodiversity is the entirety of life on the planet and the National Academies of the United States identified that it is crucial in the quest for a sustainable world (Raven 1997). From a biological perspective, indicators and indices are critical in the assessment of sustainability (Morse 2010). The variety of biological indicators are important factors in the measurement of soil health (Pankhurst et al. 1997). The early studies provide an important background as we try to exploit modern science and technology to improve soil health. It now seems appropriate to classify rhizomicrobiomics as the study and manipulation of microorganisms and their metabolic processes to optimize plant and tree growth sustainably, by integrating classical microbiology with novel molecular and mathematical approaches, as well as earth observation. Imagery is at the centre of the description of the microbiome at the cell level, but function can potentially be identified by global imagery through earth observation as identified in a recent book on an analysis of life (Lynch 2023). The concept of microscopic and global imagery offers great potential in realizing the UN Sustainable Development Goals, particularly number 15 (Life on Land). Earth observation from satellites on a global scale, which can be done at high frequency, often daily, is ideal for monitoring land use and land use change, including destruction of native forests, which are being replaced with agriculture for cattle feed and oil palm plantations (Andries et al. 2019, 2021). This is all critical in achieving the most effective land use without compromising food security.

## Origin of terminology

**Figure 1.**
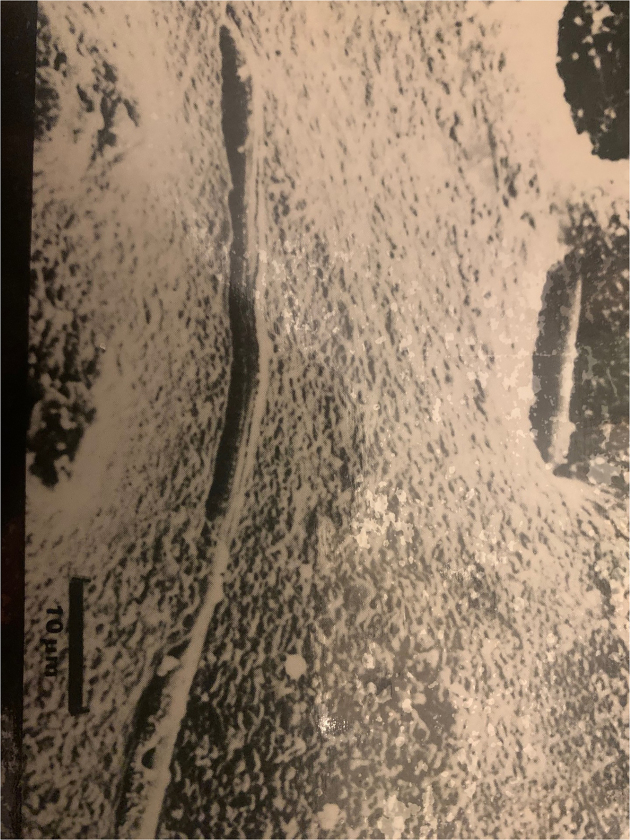
Fungal hyphae growing in wheat (*Triticum aestivum* L.) root mucilage containing bacterial cells.

The first use of the term microbiome was as a convenient ecological framework for the use of fungi in biological control systems (Whipps et al. 1988). This is confirmed by Berg et al. (2020) in their seminal review of the topic. It is defined as a characteristic microbial community occupying a reasonably well-defined habitat that has distinct physico–chemical properties. Even though some have thought fungi should be classified within a mycobiome, it seems more important for the unity of microbiology to place fungi firmly within the microbiome. The term was subsequently applied to many environments, particularly the human gut, and the journal *Science* identified the term as the breakthrough of the year in 2011 and 2013. The interdisciplinary National Microbiome Initiative was launched in Washington, DC, in May 2016. The application to animals was described by Douglas (2018). There has been a resurgence of interest in the first use of the term in plant soil systems, but in a range of areas beyond biological control, and therefore the specific term rhizomicrobiomics now seems appropriate. Traditionally, scanning electron microscopy of the rhizomicrobiome is clouded by the gelatinous material covering the organisms and it can be difficult to identify the cells within the rhizosphere (Fig. 1). Modern microscopic imagery has produced some excellent pictures of microbiomes in other environments and many of these are from the human gut in which there has been so much interest. However, the patterns are likely to be similar in all environments. Figure 2 from Lynch (2023) is one such image used to stress the importance of microorganisms in life on the planet generally. It is also important to recognize that each cell depicted will have genomes, which are factors in their function. As such, it is therefore possible to investigate the population biology of the microbial genome at the molecular level using nucleic sequencing and this identifies a much higher number of species than could be identified by culture. High-throughput next-generation sequencing (NGS) methods provide a powerful tool for high taxonomic resolution of complex microbial communities as metagenomic characterization. This has been applied effectively to branches of microbial ecology, including the analysis of wastewater treatment on biofilms (Naz et al. 2018). However, the metaproteomic and metabolomic analyses are just as important for the rhizomicrobiome (White et al. 2023). For example, the multiomics approach has been used to investigate water stress in sugar beet (Xing et al. 2023). However, with multiomics approaches come some problems. For example, some cells may not be in an active state and be merely maintained such that their function is less. Additionally, some genomes may not actively express even though they have the potential to do so. Ultimately, the key to function is to determine the components of ecosystem processes that deliver properties such as soil nutrient flow and particle aggregation and link those to the microbiomes present.

**Figure 2.**
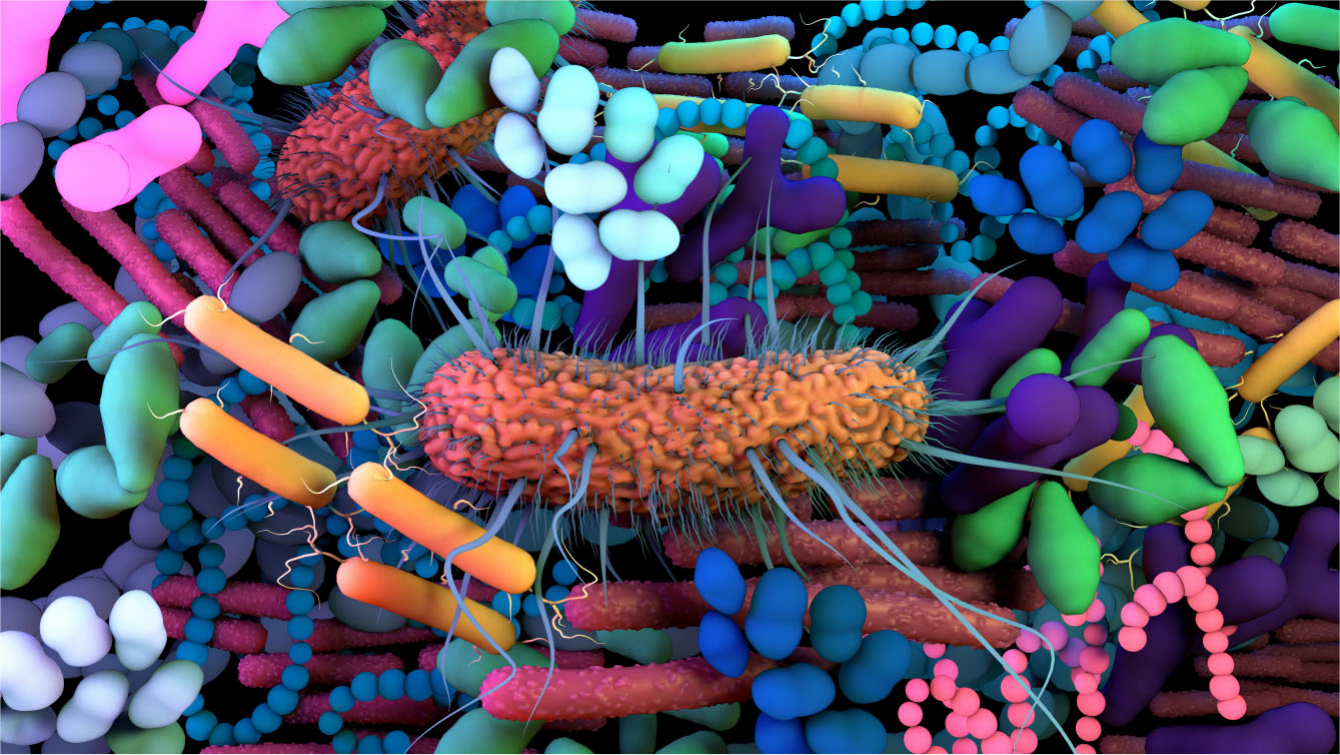
Microbiome of the human gut (Getty images).

With some exceptions (e.g. Mendes et al. 2013), the empirical analysis of rhizomicrobiomes has usually considered only free-living microorganisms. That approach ignores symbiotic and pathogenic organisms, even though the original terminology was applied primarily to biological control of pathogens. Amongst the symbionts, root nodule nitrogen-fixing bacteria and mycorrhizal fungi are significant contributors to the rhizomicrobiome, playing a vital role in the provision of nutrients to plants.

## Microbial population and community dynamics

When Pirt (1975) introduced the analysis of microbial growth dynamics in culture, he opened up the issue of organisms growing in mixed cultures as they do in nature. The concept was taken forward by Senior et al. (1976), where the population degrading the herbicide Dalapon was analysed by adding soil to a chemostat and monitoring the population that developed. In practice, a steady state of seven bacteria emerged, suggesting that small well-structured communities can carry out functions.

Substrate availability is crucial to the development of the rhizomicrobiome (Lynch 1982). The major source of substrates is derived from the root itself in a process termed rhizodeposition (Whipps and Lynch 1983) and indeed with microorganisms having the ability to stimulate carbon loss from roots (Barber and Lynch 1977). In fact, by growing plants in chambers with ^14^CO_2_ as the sole source of carbon, up to 40% of carbon captured by plants can be released as rhizodeposition products. Much of this substrate flow is polysaccharide and ends up in cells of the rhizomicrobiome. Physical model systems can be constructed to facilitate investigation of microbial dynamics in the rhizomicrobiome (Pearce et al. 1997), such as rhizodeposition. Understanding rhizodeposition allows the construction of mathematical models of microbial growth in the rhizosphere. One model constructed involved bacteria that had been introduced on seeds (Scott et al. 1995). After germination movement of substrates away from the rhizosphere into the bulk soil is by diffusion and microbial movement is mediated by carriage on the root surface. The relationship between specific growth rate and substrate concentration is described by Monod kinetics and death occurs at a specific constant rate. Matric potential has a big impact on growth, which can be followed in depth and time. The model predictions fit well with observations. Darrah (1991) points out that predation, such as by protozoa can have an impact on such models.

In soil, straw and other plant residues are important substrates for the microbiome outside of the rhizosphere, but microbial activity on it can be fed back to the rhizosphere to influence plant growth. It was shown (Veal and Lynch 1984) in a chemostat that a fungus, *Trichoderma harzianum*, associated with and an anaerobic nitrogen-fixing bacterium, *Clostridium pasteurianum*, could carry out lignocellulolysis of straw and provide nitrogen as a plant nutrient. Its requirement for anaerobiosis was supported as respiratory protection by a polysaccharide-producing bacterium, *Enterobacter cloacae*. Thus, the physical and chemical conditions for three diverse organisms could be achieved, and it can be expected that this will be achieved in the soil–plant ecosystem. Rhizomicrobiomics should therefore not just include the microbial community of the rhizosphere but also saprophytic microbial activity in soil itself, which feeds back to plants.

## Functions

Nutrient cycling is one of the major functions of the rhizomicrobiome, with nitrogen receiving much attention, especially as it relates to bulk soil, and this can depend on the energy status of bacteria and on interactions with other microorganisms, including protozoa and fungi. Under the influence of root-derived carbon, bacteria are temporarily not energy-limited and start to mineralize nitrogen from organic matter, which will be immobilized by increased bacterial biomass. This depletes the pulse of carbon from roots and the bacteria can be consumed by amoebae attracted to the site. As the protozoa digest bacteria, it releases part of the bacterial nitrogen as ammonium at the root surface, where it can be taken up by the root. This source of ammonium can be supplemented by fungal decomposition of organic matter and energy-limited bacteria in the bulk soil. This can then diffuse towards the root to be absorbed as ammonium or after nitrification as nitrate. If the plant is a legume hosting symbiotic *Rhizobium* spp. bacteria on and in its roots, a further nitrogen pool is generated for the plant. This pool can also be supplemented by any free-living bacteria around roots.

**Figure 3.**
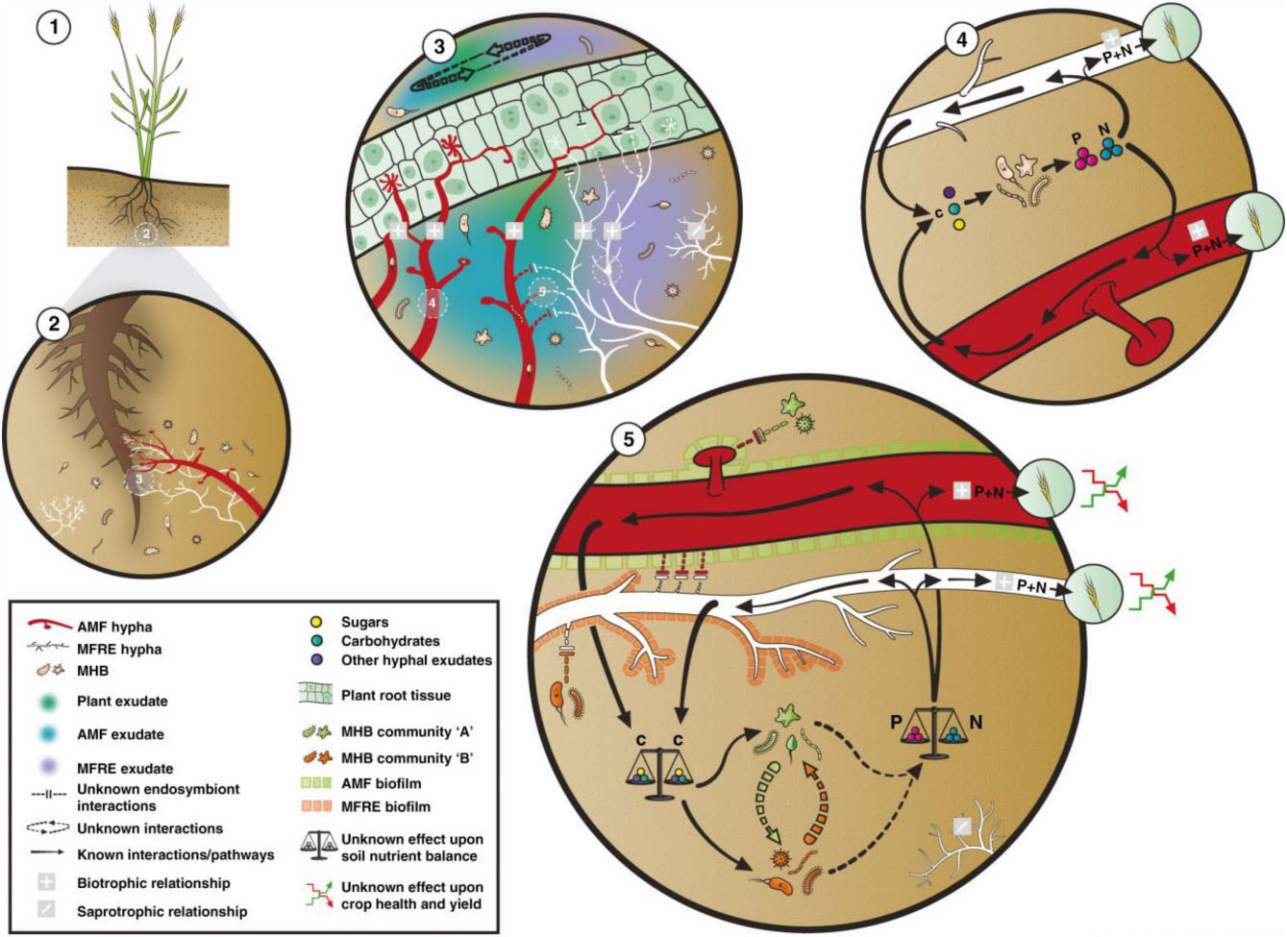
Step-wise overview of putative multipartite interactions between plant roots, mycorrhizosphere microbial communities and arbuscular mycorrhizal (AM) fungi and Mucoromycotina fine root endophyte (MFRE) fungi. 1. A sessile plant. 2. Exclusive MFRE fungi (white hypha) interactions are rare, often forming simultaneous associations alongside AM fungi (red hypha); diverse bacterial populations associate with fungi in and around the hyphae and this is termed the mycorrhizosphere. 3. Putative/unknown overview (dashed lines) of complex plant root and fungal hyphal exudate interactions. 4. Overview of potential bacterial interactions with both AM and MFRE fungi in terms of nutrient mineralization and transfer to host plant. 5. Summary panel of fundamental questions from mycorrhizal bacterial–fungal interactions (BFIs) including analysis of putative biofilms, effects of bacterial populations on nutrient mineralization and mycorrhizal function, bacterial–bacterial (B-B) interactions, fungal–fungal (F-F) interactions and BFIs (Williams et al. 2024). Reproduced with the permission of the authors and Oxford University Press.

Similar arguments can be applied to the cycling of phosporus in the rhizomicrobome. The first studies on the role of bacteria in promoting the uptake of phosphorus by plants were carried out at Letcombe and CSIRO Adelaide (Barber et al. 1976). Plants were grown axenically in nutrient culture in the presence or absence of a mixed population of bacteria and uptake studied using phosphate labelled with radioactive ^32^P. Both symbiotic and free-living microorganisms can influence the uptake of nutrients by plants, with most studies concentrating on nitrogen and phosphorus. Symbiotic associations include those with mycorrhizal fungi, which can be around roots (ectosymbionts) or within roots (endosymbionts). Both symbiotic and free-living microorganisms can influence the uptake of nutrients by plants, with most research focussing having on nitrogen and phosphorus. Much of the pioneering work on mycorrhiza was by Jack Harley, first in Sheffield and then in Oxford (Harley and Smith 1983). The work in Sheffield was continued by David Read, while other centres were led by Barbara Mosse in Rothamsted, UK, and Jose-Miguel Barea in Grenada, Spain. When I was appointed by Jack Harley to Letcombe in 1971, there had been little attempt to integrate studies of rhizosphere bacteria with those of fungi, and therefore he advised that in searching for function in soil, both bacteria and fungi should be considered. Barea et al. (2002) identified, the importance of a mycorrhizosphere improving soil plant fitness and soil quality. Bacterial partners of the of the fungal hyphosphere (also termed mycorrhiza helper bacteria, MHB) can facilitate spore germination and mycelial growth of mycorrhizal fungi (Sangwan and Prazamna 2002). In addition, MHB can influence plant growth and biofilm formation, via chemical communication. These fascinatingly complex bacterial–fungal interactions (BFIs) play a vital role in nutrient cycling and overall ecosystem functioning (Fig. 3; Williams et al. 2024).

Bacteria can influence plant growth positively or negatively and can be heavily dependent on plant cultivar, as demonstrated, e.g. during the wheat breeding programme in the Pacific Northwest of the United States in relation to pseudomonads around roots (Elliott and Lynch 1984). Older wheat cultivars bred prior to the extensive use of nitrogen fertilizers had rhizomicrobiomes that were more capable of supplying their own nitrogen from fixation. The rhizomicrobiome can influence plants with the provision of plant growth regulators, and for many years, there has been much study of plant growth-promoting rhizobacteria (PGPR), both by natural processes but also with the potential to use them by inoculation. Several products appeared on markets, but none have been a great commercial success. Not all plant growth regulators are beneficial and most exhibit a dose-response curve of benefit at low concentrations while being harmful at high concentrations. For example, ethylene produced endogenously in the plant stimulates growth in many species, but when ethylene is produced by fungi in wet anaerobic soils, it retards root growth (Lynch 1972). Other growth regulators within microbial sources are indole acetic acid, gibberellic acid, and abscisic acid, all of which have had limited commercial application and success as interventions.

Some substances that are produced microbially in the rhizosphere or bulk soil can be phytotoxic without being growth regulators. These components of the community include deleterious rhizobacteria. They can often be a problem in wet anaerobic soils and can be associated with crop residues as substrates, with the substances diffusing to the roots. For example, acetic acid is a product of cellulolysis by anaerobic bacteria but can be catabolized by aerobes, achieved practically by ploughing the soil (Lynch 1978).

The microbiome, when disrupted, can include pathogens such as fungi, bacteria, and nematodes. These can often be controlled by agrochemicals. Naturally, they can have their impact reduced by antagonist organisms, but there has also been considerable success of exerting this effect by inoculation with biocontrol organisms. To achieve this commercially, the biocontrol agents need rigorous testing to ensure there are no harmful side effects and this can be expensive. There have been some successful products, usually by small niche companies, but they have been dwarfed by the agrochemical industry. They have the potential to be more environmentally friendly than chemical loading of the environment with pesticides such as highlighted by Rachel Carson (1962) in the use of the insecticide DDT.

As Rachel Carlson highlighted the danger of pesticides and other pollutants like heavy metals in the environment, the opportunity arises for phytoremediation. This is an ongoing option, such as a recent paper on the sustainability of soil copper remediation (Espada et al. 2024), but thus far there has only been commercial development on a limited scale. The rhizomicrobiome is likely to be crucial in phytoremediation. The fungus *Trichoderma harzianum* has been an agent of plant growth promotion while also able to bring about remediation. In a joint venture company between Cornell and Surrey Universities, this was termed phytobial remediation (Harman et al. 2004). The concept applies equally well to trees as crop plants. For example, *Trichoderma harzianum* could promote the growth of crack willow in clean and contaminated soil (Adams et al. 2007).

One of the major components of rhizodeposition is polysaccharides. These gels usually have an important role in stabilizing soils and giving them structure (Lynch and Bragg 1985). Rotation of crops to generate different rhizomicrobiomes in soil can often be an effective means of preventing soil degradation. Often, very good structures can be generated by perennial species, including trees. Soil stability is a crucial element of sustainability.

## Gene transfer

One of the opportunities in genetic modification is to enhance sustainability while not compromising existing ecosystems. In some of the earliest molecular genetic studies of rhizomicrobiomes attention was focussed on a bacterium *Agrobacterium tumefaciens*, which is the cause of crown gall disease of fruit trees (almond, peach, rose, and cherry). Plasmids are mobile genetic elements and the virulence genes of *A. tumefaciens* are located on a large (200 kb) plasmid (the Ti-plasmid) and the T-DNA is transferred to the plant cell (Kerr and Tate 1984). *Agrobacterium rhizogenes* strain 84 is a non-pathogenic isolate that produces the antibiotic agrocin 84 coded for on the plasmid pAg 396 and can be taken up by the pathogen to kill it. Strain 84 had some success as a biocontrol agent following inoculation. The Ti-plasmid became one of the major agents used in genetic modification of plants with much of the work being done in the laboratory of Marc van Montagu at the University of Gent in Belgium. In Gent, the importance of the total rhizosphere bacterial population was also recognized.

It became clear that plasmids could be potential agents for the flow of genes in microbiomes. In some preliminary observations in India, it was found that in wastewater with heavy metals, antibiotic resistance and heavy metal resistance were coded for on the same plasmids, being one factor in the widespread occurrence of antibiotic resistance in humans. To investigate this amongst organisms of the rhizomicrobiome, sterile microcosms with clay loam, sandy loam, and sandy soils were constructed and intrageneric and intergeneric broad-host-range plasmids, belonging to IncP (RP4 and pUP1102) and IncC (R57.b), derived from *Escherichia coli* and *Acinetobacter calcoaceticus*, were studied. High-frequency exchange was found to organisms, which included the free-living nitrogen fixer *Azotobacter chroococcum* and the symbiotic nitrogen fixer *Rhizobium meliloti*, especially if the soil was amended with nutrients (Naik et al. 1994).

Transgenic plants and transgenic microorganisms could potentially influence microbial diversity in soil. A range of ecological theories, such as the Shannon-Weaver diversity index, and molecular techniques can be used to investigate this (Lynch et al. 2004). However, little evidence of horizontal gene transfer has ever been observed in field soil from transgenic plants or microorganisms, but this could be underestimated because of low detection limits. This is not to be confused with the widespread incidence of antibiotic resistance, which has occurred because of excessive over-prescription and some of which can leak into the environment.

Many constructs of biocontrol bacteria for the rhizosphere have antibiotic-resistance markers. It was therefore of concern that, if released into the environment, they might interact with the native rhizomicrobiome to influence populations and functions. The first field trials to test this possibility in the United Kingdom were carried in 1993 out in Littlehampton in West Sussex with seed and spray applications to a wheat crop (De Leij et al. 1995), with similar studies carried out at Wytham in Oxford to investigate the impact on the phytosphere (the combined arena around roots and shoots) of sugar beet (Thompson et al. 1995). The genetically modified microorganism (GMM) chosen was a plasmid-free *Pseudomonas fluorescens* SBW 25. The first marker gene cassette contained the *lacZY* genes, which could be detected by lactose utilization and the utilization of the chromogenic substrate *X*-gal to yield a blue colour. The second marker cassette, *aph-xylE*, encoded resistance to kanamycin (*aph*) and degradation of catechol to yield a yellow colour. The effects were compared with those of an unmodified wild-type bacterium. The GMM could colonize wheat and sugar beet plants from seeds and survived on decaying wheat roots for up to 300 days after harvest, but after 3 years, the GMM was no longer detected in soil. With wheat, the GMM spread to a vertical depth of 45 cm and laterally to 2 m. No transfer of chromosomal markers to the indigenous bacteria was detected but mercury-resistant plasmids were detected in the GMM from the native population. Models of microbial growth and dispersal, such as Scott et al. (1995), are highly relevant to such releases. Environmental impact can have several components, such as impact on enzyme activity. In studies with the biocontrol agent *Pseudomonas fluorescens* F113, which produces the antibiotic 2,2-diacetylphloroglucinol, there was no damage to soil enzyme activities or soil chemistry in real soil caused by the agent (Naseby et al. 1998).

## New directions

**Figure 4.**
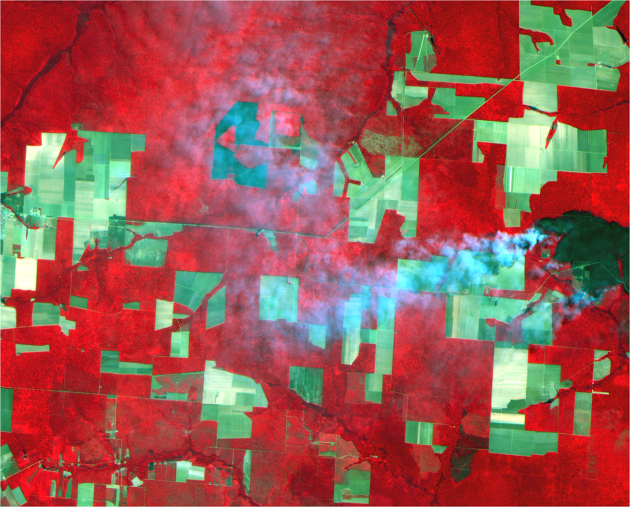
Clearance of Amazon rain forest by illegal logging and fire, with associated cropping.

The 21st century has opened the opportunity for molecular biology techniques to characterize the much more extensive analysis of populations than had been available with conventional microbial observation and isolation techniques. This is particularly so for bacteria and there is still much to be done on the contribution of other microbial groups to the total biome and how they interact with the bacteria. Together, they bring about the function of the rhizosphere, which determines how plants and trees grow. The most direct measurement therefore of function is the health and biomass of crops and forests. On a small scale, this can be assessed by direct observation and measurement, but this can be tedious and expensive on a large scale. With the rapid advance of satellite technology at increased resolution, even as low as 30 cm, and reduced costs, earth observation is a good option to study how the rhizomicrobiome affects plant health and production as well as the capacity to capture carbon in the fight to avoid climate change. An obvious use of the technology is tracking illegal logging or burning of trees to make way for crops such as soya to feed animals in the beef industry in places like Amazonia (Fig. 4). It is crucial that there is international consensus on the monitoring, reporting, and verification (Lynch et al. 2013). Earth observation can also be used to assess plant health. For example, nutrient or water deficit stress, or pests and diseases, will often cause yellowing of leaves, and the chromophore change can be readily assessed by satellites on a regular basis, even daily, on a large scale, which gives more scope in management of threats and opportunities, including the risks and benefits of ecotourism. Satellite technology has already been exploited commercially in precision agriculture, such that fertilizer applications can be more specifically targeted in a field rather than applying blanket coverage in some areas where applications are not needed. This is both economically sensible and more sustainable. One recent study has also assessed biodiversity change associated with vegetation in the Surrey Hills of England (Andries et al. 2021). Coupling of earth observation along with *in situ* microscopic evaluation of microbial populations and their activity is an appropriate way forward for rhizomicrobiomics in an integrated analysis of land use and the generation of a sustainable planet. This has already been proven highly successful in another arena of microbial ecology, aquatic microbiology, where earth observation has been used by Rita Colwell and colleagues to study the plankton blooms carrying the bacteria in Bangladesh, which carry the cause of cholera, *Vibrio cholerae*, and predict when disease outbreaks will occur in relation to climate judged by sea surface temperature and sea height (Lobitz et al. 2000). The possibility of using this approach in the application of this new discipline of rhizomicrobiomics has already been flagged up (Beatty et al. 2021), and eDNA of soils to determine the microbial communities seems like a good prospect (Skidmore et al. 2022).

## Discussion

In recent years, there has been a considerable increase in interest in microbiology, not the least of which was generated by the global COVID pandemic. However, there has also been considerable press coverage of gut health, with the microbiome being mentioned frequently. It seems that the metabolic activity of the gut microbiome can send chemical signals to the brain and influence Parkinson’s and Alzheimer’s diseases (King 2024). It is perhaps ironic that the concept started with the analysis of the microbiomes of plants, especially how they might have their diseases controlled by antagonists within the microbiome. In the gut, it has been shown that gut health can sometimes be improved by probiotics such as *Lactobacillus* spp. or by faecal transfer from healthy guts. There is now an opportunity to use synthetic microbial communities (SynComs) to achieve this, according to Van Leeuwen et al. (2023). In many ways, this is analogous to the biological control of plant diseases using microbial strains that have been isolated from healthy soils. Indeed, soil health is a key outcome to the application of the concept of rhizomicrobiomics. This can also ultimately improve global food security, which will be compromised increasingly. With ∼40% of the plant’s photosynthate being released as rhizodeposition products (Whipps and Lynch, 1983), this large pool of carbon is often disregarded in compiling global carbon budgets and therefore not fed into the concept of carbon trading. The rhizomicrobiome therefore feeds into the global challenge of climate change, as well as food security. We will need to move towards more cropping for food and change our dietary habits and lifestyles (Sadhukhan et al. 2020) to realize the UN Sustainable Development Goals. The concept is also relevant to wilding (Tree and Burrell 2023), where land is used to improve lives as an ecosystem service, including ecotourism, which can often sustain rural communities. Hopefully, research activity will expand in this arena by adding novel technologies such as artificial intelligence and machine learning, as well as modern molecular biology and mathematical modelling, to integrate with the extensive pioneering work over more than a century. This will hopefully lead to a more sustainable world. Societies such as Applied Microbiology International are now active in promoting this arena, and it is important to establish international discussion groups to promote this, such as Microbes-4-Climate, established in February 2024, to address climate change risks for biodiversity in agricultural and forestry ecosystems.

## Data Availability

All relevant data are contained within this article.
